# Community Pharmacists’ Experiences and Perception about Transitions of Care from Hospital to Home in a Midwestern Metropolis

**DOI:** 10.3390/pharmacy9040193

**Published:** 2021-11-27

**Authors:** Rachel K. Vossen, Yifei Liu, Peggy G. Kuehl

**Affiliations:** 1 Community Pharmacy Residency Program with Balls Food Stores, The University of Missouri-Kansas City School of Pharmacy, Kansas City, MO 64108, USA; rvossen@shoppersdrugmart.ca; 2 Division of Pharmacy Practice and Administration, The University of Missouri-Kansas City School of Pharmacy, Kansas City, MO 64108, USA; kuehlp@umkc.edu

**Keywords:** community pharmacist, transitions of care, hospital discharge, pharmacist services, health communication, medication-related problems

## Abstract

Objectives: (1) To describe the experiences of community pharmacists in transitions of care (TOC) from hospital to home in a Midwestern metropolis; and (2) to develop instruments to measure perceived importance of TOC activities. Methods: Survey items were developed, including a six-item instrument to capture perceived importance of TOC activities. The items were piloted to examine face validity before dissemination to 310 community pharmacists. Descriptive statistics were reported. Principal component analysis and reliability analysis for the six-item instrument were performed to assess construct validity and Cronbach’s alpha, respectively. Results: The response rate was 37% (*n* = 118). The majority of community pharmacists estimated that they learned of a patient’s discharge on less than 10% of the occasions. There were 76 cases in which the discharged patients experienced either a prescription- or medication-related problem. For the six-item measurement of perceived importance, one component was yielded and all items loaded on the component with high values, which confirmed construct validity. The Cronbach’s alpha for these six items was 0.941, indicating high reliability. Conclusions: A large communication gap existed for community pharmacists to receive patient discharge information. The six-item instrument to measure perceived importance of TOC activities was valid and reliable.

## 1. Introduction

Transitions of care (TOC) occur when patients move from one health care setting to another [1]. It is estimated that poorly coordinated transitions from hospital to other settings in the U.S. cost USD 12 to 44 billion per year [2]. A portion of this cost is related to medications. For example, 19–23% of patients discharged experience adverse drug events within five weeks [3,4], and 40–90% of patients discharged have medication discrepancies [5,6,7,8]. Of those with medication discrepancies, 25–30% can potentially develop serious morbidity [5,8,9]. Therefore, in the Hospital Chapter of the 2021 National Patient Safety Goals, the Joint Commission on Accreditation of Healthcare Organizations (JCAHO) highlights the criteria for safe medicine use [10].

Studies have reported the benefits inpatient pharmacists can provide regarding medication reconciliation and care coordination [5,6,7,8,9,11,12]. Inpatient pharmacists have greatly reduced discrepancies upon discharge and high-risk medication errors [6,7,12]. However, in addition to inpatient pharmacists, care coordination efforts are also needed from community pharmacists. International studies have suggested a role for community pharmacists. In Australia, Roughead et al. found that patients visited their community pharmacy sooner than their general practitioners after discharge [13]. In the U.K., Urban et al. revealed community pharmacists rarely received discharge information from providers, and when they did, it was often inadequate to resolve discrepancies [14]. However, in Canada, McCarthy et al. reported the usefulness of discharge summaries for community pharmacists [15].

Evidence is emerging in the U.S. to establish a role for community pharmacists in TOC [16]. For instance, two studies reported that community-pharmacy-based TOC programs significantly reduced 30-day readmissions [17,18]. In particular, after examining a sample of 1,219 patient encounters, Shaver et al. concluded that those who were in a TOC program had 67% lower odds of 30-day readmission [18]. Nevertheless, there are barriers for community pharmacists in post-discharge patient care, such as communication issues, organizational resources, and patient characteristics [19,20]. In the study of Farley et al., discharge information was sent to community pharmacists, but no additional reduction in discrepancies was found [12]. It was hypothesized that there was little incentive for community pharmacists to remove outdated medication prescriptions. Therefore, specific barriers preventing effective pharmacist integration and action need to be identified. 

Six models have been developed across different settings to guide TOC in the nursing literature [21]. For example, Naylor et al. have established the Transitional Care Model, a team-based model led by an advanced practice nurse to improve TOC from hospital to home [22,23,24,25,26]. A commonality among these models is to promote and optimize patient-centered care. To further strengthen the role of community pharmacists in TOC, peer perspectives and experiences are needed. Yet, valid and reliable instruments to capture community pharmacists’ perception about TOC activities for optimal patient care are lacking. The objectives of this study were (1) to describe experiences of community pharmacists in TOC from hospital to home in a Midwestern metropolis; and (2) to develop instruments to measure perceived importance of TOC activities. This study was not meant to examine the hospital discharge process, but to describe the experiences and opinions of community pharmacists in TOC.

## 2. Materials and Methods

Based on the literature and our experiences, we developed survey items to examine community pharmacists’ experiences in TOC, and perception about how important TOC activities are for optimal patient care (Supplementary Materials). We made two assumptions when designing the survey: (1) the beginning of TOC occurs when hospital-based physicians discharge a patient; and (2) community pharmacists may not be fully utilized in TOC communications. 

As mentioned earlier, the Transitional Care Model is a TOC model for nursing practice. Nevertheless, it can be adapted for community pharmacy practice. A previous version of this model incorporated nine components, whereas the latest version further develops them into ten components [25,26]. We proposed placing the survey items (excluding pharmacist characteristics measurements) under two components: (1) Collaborating [25], or Collaborating with Patients, Caregivers, and Team [26]; and (2) Assessing/ Managing Risks and Symptoms [25], or Managing Symptoms and Other Risks [26] (Table 1). Perceived importance is an individual state construct that is theorized to impact the behavioral intention and the behavior itself [27]. Perceived importance of TOC activities was proposed to be under the component of collaborating, because this construct may drive community pharmacists to communicate with hospital-based physicians. 

Survey items included questions which asked community pharmacists to estimate a percentage of occasions that they were notified of a patient’s discharge. They were also asked to recall their most recent encounter with a discharged patient and then to identify the types of interventions they performed. Interventions included identifying both prescription-related problems (i.e., incomplete or unclear instructions, Schedule-II prescription not valid, mismatch between quantity prescribed and days therapy, issue with prescriber identity or verification, or formulary issue) and medication-related problems (i.e., wrong drug, wrong dose/route, drug interactions/adverse reactions, duplicate therapy, untreated indication for therapy, medication adherence) [28]. Subsequent questions evaluated the number of times they attempted to contact the prescriber, barriers encountered, and estimated response time. Furthermore, we developed six items (Table 2) to capture the construct of perceived importance of TOC activities for optimal patient care (5-point Likert scale, 1 = Strongly Disagree, 5 = Strongly Agree).

The survey was piloted with five subjects before dissemination. Based on feedback, changes were made, and the revised survey was piloted again with a separate group of five subjects before dissemination. Pilot tests were used to examine face validity of survey items, and invalid items were excluded.

Community pharmacists were identified and included if their contact information was on a pre-existing email list from the University of Missouri—Kansas City School of Pharmacy (*n* = 319). This list included pharmacists who were affiliated with the School of Pharmacy and who practiced in community pharmacy settings in Kansas City. The cross-sectional survey was administered using an online survey tool, SurveyMonkey^®^ (Momentive Inc., formerly SurveyMonkey Inc., Palo Alto, CA, USA). Survey links were generated and emailed to participants. Surveys were available for 30 days after initial distribution, and reminder emails were sent two additional times throughout the study period. 

Descriptive statistics such as the mean, standard deviation, and frequency were reported. Principal component analysis and reliability analysis for the six-item measurement of perceived importance were performed to assess construct validity and Cronbach’s alpha, respectively. All data analyses were conducted using IBM SPSS Statistics (IBM Corporation, Armonk, NY, USA). 

## 3. Results 

**Figure 1:**
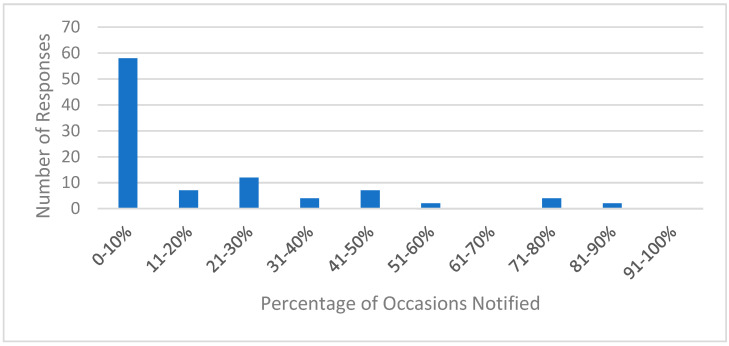
Percentages of occasions when community pharmacists were notified of a patient’s discharge.

A total of 118 community pharmacists completed the survey, with a response rate of 37% (118/319). They had practiced an average of 15.8 years, were predominantly female (60.2%,) and were from a variety of settings (Table 3). The majority estimated that they learned of a patient’s discharge on less than 10% of the occasions (Figure 1). Regarding the information source of a patient’s discharge, multiple answers were allowed. Overall, community pharmacists learned about a discharge most often verbally from a patient or caregiver (*n* = 69). They also learned about it from the written prescription (*n* = 36), patient or caregiver providing discharge papers (*n* = 30), patient/caregiver asking questions regarding discharge (*n* = 13), or a physician or hospital communication (*n* = 13). When they learned of a patient’s discharge but did not have access to the discharge medication list, 40 community pharmacists asked about other medication changes. 

Community pharmacists reported an average of 1.6 prescription-related problems and 1.3 medication-related problems for the most recently discharged patient. Incomplete or unclear directions and issues with prescriber identity were the two primary prescription-related problems (51.5%, *n* = 53; and 45.6%, *n* = 47, respectively); while drug interactions or adverse events, and duplicate therapy were the two primary medication-related problems (31.1%, *n* = 32; and 29.1%, *n* = 30, respectively) (Table 4). Multiple answers were also allowed for both of these questions.

Overall, community pharmacists reported 76 cases in which discharged patients experienced either a prescription- or medication-related problem. Community pharmacists attempted to contact the prescriber in 72 of these cases. However, in 50 cases, they were not able to reach the prescriber on the first try for the following reasons (multiple answers were allowed): unable to directly contact prescriber (*n* = 32); could not reach prescriber outside of office hours (*n* = 30); or had missing (*n* = 21), inaccurate (*n* = 16), or illegible (*n* = 7) contact information. Community pharmacists reported an average of 2.3 days to receive a prescriber response, and in 17 cases did not receive a response at all. When asked if their most recently discharged patient received all medications prescribed at discharge, 36 community pharmacists reported “no” with the following reasons (multiple answers were allowed): a prescription problem that could not be resolved at the time of pick-up (*n* = 23), insurance restrictions (*n* = 16), cost of the medication (*n* = 9), patient not expecting a new medication (*n* = 3).

For the six-item measurement of perceived importance, the principal component analysis showed only one component was yielded, and all items loaded on the component with high values, which confirmed construct validity (Table 5). The Cronbach’s alpha for these six items was 0.941, indicating high reliability. In addition, the average ratings of community pharmacists were 3.9 for Item 1, 4.2 for Item 2, 4.0 for Item 3, 3.9 for Item 4, 4.0 for Item 5, and 3.9 for Item 6. 

## 4. Discussion

This study contributes to the understating of specific barriers for pharmacists in TOC, and pharmacists’ perceived importance of specific TOC activities. Our findings indicate a large gap in communication with regard to patient discharge information. Most community pharmacists learned of a patient’s discharge infrequently and from patients or caregivers, rather than official communication between healthcare settings. Although this gap is consistent with the literature, we identified being unable to directly contact the prescriber and outside of business hours as two primary barriers. These barriers add to the existing knowledge, as Freund et al. reported two common barriers to be no discharge medication list and patient absence in consultation [19]. In addition, it took over two days to receive a prescriber response. Without community pharmacists’ input, it would be difficult to achieve desirable outcomes in TOC, especially for medication management. This study is a necessary reminder that the voice of the community pharmacists must be heard. Furthermore, our findings reveal that while community pharmacists perceived TOC activities to be important, the communication barriers existed to a large extent and were not fully understood. 

Based on our findings, it appears there is a major opportunity to use community pharmacists to reduce prescription- and medication-related problems associated with TOC. Kennelty et al. reported that receiving hospital medication discharge list and stop orders for discontinued medications would be helpful for community pharmacists [20]. Of note, it is possible that hospital-based physicians are not the ones actually sending the information, or they are unsure of who completes this task. In this study, even without consistent notification or availability of discharge plans and medication lists, community pharmacists were able to identify prescription- or medication-related problems in 73.8% of recently discharged patients. 

Potential solutions for bridging the communication gap and utilizing community pharmacy services have been proposed. Luder et al. found that when a community pharmacist facilitated outpatient discharge follow-up, they were able to significantly reduce hospital readmission rates [17]. As various collaborative care models have been tested in the U.S., there is a need for further research to define best practices, who should be included, and financial avenues to help support these services. Our study can serve as a stepping-stone to better understand needs, gaps, and opportunities to engage community pharmacists in TOC and improve patients’ health outcomes. Future efforts should explore potential mechanisms to link provider groups for seamless information transfer, determine an optimal financial scenario including both inpatient and community pharmacists, and track patient outcomes of interventions, specifically readmission rates.

In addition to describing community pharmacists’ experiences in TOC, this study made a significant contribution to instrument development for the construct of perceived importance of TOC activities. The six-item measurement passed the examinations of face validity, construct validity, and reliability, in addition to collecting valuable peer experience and perception information. Validating the construct of perceived importance was the first step toward utilizing it in theory building. 

Using the Transitional Care Model, we proposed placing perceived importance under the component of collaborating. Namely, collaborating would be a higher-level construct, and perceived importance could be a predictor for collaborating. According to the latest version of the model, three criteria need to be met for the use of the component: (1) “Collaborates with care team, occurs within site”; (2) “Collaborates with care team, occurs across sites”; and (3) “Assures direct communication occurs between hospital and primary care clinicians” [26]. The three criteria can be modified for community pharmacy practice as follows: (1) Collaborates with care team, occurs within the community pharmacy; (2) Collaborates with care team, occurs between the hospital and community pharmacy; and (3) Assures direct communication occurs between hospital-based physicians and community pharmacists. For instance, future researchers can design a longitudinal study in which perceived importance of TOC activities is measured in the baseline survey, and collaborating is measured in the follow-up survey. Such a study would not only be able to assess the criterion-related validity of perceived importance given the construct of collaborating, but also to quantify the usefulness of perceived importance. As for other variables in our survey, future researchers can select some of them as control variables to predict collaborating along with perceived importance. Again, we only conducted a cross-sectional study for the initial step to validate the construct of perceived importance, and future studies can further utilize this validated construct. 

Another direction for future research can be a mixed-method approach to include community pharmacists, other health care professionals, patients and care givers, and hospitals’ policies for a comprehensive understanding of TOC communications. Other health care professionals include hospital-based physicians, primary care physicians, nurses, physician assistants, patient navigators, and so on. In particular, if the health care professionals work together in the same care team, their insights would be helpful toward understanding each other’s needs, communication gaps and barriers, and opportunities for community pharmacists to improve patient discharge communication. For instance, the patient navigators may know why the community pharmacists are not notified of a patient discharge. Hospitals’ policies may also have communication procedures for TOC. 

In addition to this study, we developed a survey for hospital-based physicians, and administered the survey in the same way. Physicians were identified from a pre-existing email list of hospitalists or medical residents associated with the University of Missouri—Kansas City School of Medicine and provided inpatient care at a hospital in Kansas City (*n* = 225). However, due to a very low response rate of 17% and no hospitalist respondents, it was determined that the results were not fit to be reported. A common lesson learned from this study and our physician survey is that three contacts with survey recipients were not enough to achieve a high response rate, and we should have sent out more reminders or used incentives.

There were five limitations in this study. First, the response rate was only 37%, and non-response bias might have occurred. Regardless, we learned of the experiences of 118 community pharmacists who provided information on patients who had recently been discharged. We did not contact nonrespondents for why they did not respond. On one hand, some nonrespondents might be less engaged in TOC, so they did not respond. On the other hand, some nonrespondents might have a heavier workload, so they did not have time to respond. Second, we relied on convenience sampling, and community pharmacists affiliated with a school of pharmacy often serve as preceptors and may be more engaged in developing innovative pharmacy services or spending time with students. The sample might not be representative of community pharmacists in general. In addition, the sample was in one Midwestern metropolis, thus not generalizable to non-Midwestern regions in the U.S. Third, we only asked about the most recent discharged patient encounter. Responses could have been different regarding other patient encounters. However, we chose to specify their last encounter as they should remember that one the best, and we would hopefully get a more representative sample of cases, rather than get the worst case they had ever encountered. Fourth, because community pharmacists did not routinely learn of patient discharges, it was possible that the percentage of occasions they reported might be inaccurate. Regardless, the point that they were not routinely notified still stands. Fifth, it was not possible for us to know if any medication reconciliation was performed within the hospital before the patients were discharged. This might have affected the reported number of prescription- or medication-related problems. 

## 5. Conclusions

In conclusion, the study identified a large communication gap for community pharmacists to receive patient discharge information. Moreover, the six-item instrument to measure perceived importance of TOC activities was valid and reliable. 

## Figures and Tables

**Table 1:** Proposed mapping between survey items and two components of the Transitional Care Model.

Component ^a^	Survey Item (Supplementary Materials)
Collaborating [25]; Collaborating with Patients, Caregivers, and Team [26]	The mechanism to learn of a patient’s discharge (Q5), and medication changes (Q6–Q7); The frequency to be notified of a patients’ discharge (Q18) Perceived importance of TOC activities (Q19)
Assessing/Managing Risks and Symptoms [25]; Managing Symptoms and Other Risks [26]	Prescription- and medication-related problems, and relevant interventions (Q8–Q15) Patient receiving prescribed medications upon discharge (Q16–Q17)

^a^ Each component is provided with two names, one from a previous version of the Transitional Care Model [25], and the other from the latest version [26].

**Table 2:** The six-item measurement for perceived importance of TOC activities.

Item	Instrument ^a^
	How important to optimal patient care is each of the following items?
Item 1	Community pharmacist is notified when one of their patients is discharged.
Item 2	Community pharmacist receives discharge documentation/ updated medication list upon patient discharge.
Item 3	Community pharmacists have access to information needed to identify medication and discharge errors.
Item 4	Community pharmacists have access to information needed to answer patient questions about discharge plan or medications.
Item 5	Community pharmacists can directly contact the original prescriber to resolve prescription or medication problems
Item 6	Community pharmacists notify primary care providers when patients do not receive discharge medications

^a^ 1 = Strongly Disagree, 3 = Neither Agree nor Disagree, 5 = Strongly Agree.

**Table 3:** Characteristics of community pharmacists who participated in the study (*n* = 118).

Characteristic	Value
Years in Practice, mean ± SD (range)	15.8 ± 12.3 (1–41)
Gender, no. (%)	
Female	71 (60.2)
Male	47 (39.8)
Practice Setting, no (%)	
Independent Pharmacy (<4 stores under same ownership)	32 (27.1)
Small Chain Pharmacy (4–10 stores under same ownership)	8 (6.8)
Large Chain Pharmacy (>10 stores under same ownership)	32 (27.1)
Mass Merchandiser Pharmacy (e.g., Wal-Mart, Kmart)	9 (7.6)
Supermarket Pharmacy (e.g., Price Chopper, Hy-Vee)	26 (22.0)
Other	11 (9.3)

**Table 4:** Prescription- or medication-related problems identified by community pharmacists (*n* = 103).

Problems	No. (%)
Prescription-related problems ^a^	
Incomplete or unclear directions	53 (51.5)
Issues with prescriber identity or verification	47 (45.6)
No prescription problems identified	34 (33.0)
Mismatch between quantity prescribed and days therapy	23 (22.3)
Discrepancy among medication list, prescription written, and patient’s old medications	9 (8.7)
C-II prescription not valid	8 (7.8)
Medication-related problems ^a^	
Drug interactions/adverse reactions	32 (31.1)
Duplicate therapy	30 (29.1)
Wrong dose/route	19 (18.4)
Adherence (patient unable or unwilling to adhere to treatment)	17 (16.5)
Wrong drug	8 (7.8)
Untreated indication for therapy	6 (5.8)
Suboptimal drug therapy	1 (1.0)
Drug not needed	1 (1.0)
Other	2 (1.9)

^a^ Multiple answers were allowed.

**Table 5:** Principal component analysis for six-item measurement of perceived importance.

Item	Component 1
Item 1	0.830
Item 2	0.858
Item 3	0.920
Item 4	0.955
Item 5	0.889
Item 6	0.818

## Data Availability

Data sharing is not applicable to this article.

## Supplementary Materials

The following are available online at https://www.mdpi.com/2226-4787/9/4/193/s1, Supplementary Materials: Pharmacist Survey Regarding Patient Discharge.
