# Supplementary material for: Community Pharmacists’ Experiences and Perception about Transitions of Care from Hospital to Home in a Midwestern Metropolis
Source: Pharmacy (Basel). 2021 Nov 27;9(4):193. doi: 10.3390/pharmacy9040193 (PMC8708755; doi:10.3390/pharmacy9040193)
Supplement: Supplementary file 1 [file pharmacy-09-00193-s001.zip › pharmacy-1425014-supplementary.pdf]

Pharmacist Survey Regarding Patient Discharge

Currently there are no data describing the frequency and completeness of information shared with community pharmacists regarding patients recently discharged from the hospital.

This survey is to ask you for details regarding the most recently hospitalized and discharged patient for whom you provided care. You will also be asked to provide an estimated frequency of various care steps related to discharged patients and to rate how important you believe these steps are to providing optimal patient care. By collecting these data, we will obtain information that can be used to characterize the nature of communication between hospital providers and the community pharmacist and also between the community pharmacist and the patient's primary care provider with regard to patient care in the transition from hospital to home. By comparing this information with the opinions of hospital and primary care physician providers, we believe we can make a contribution to this area of patient care that is underappreciated and often unrecognized.

***1. By selecting "Yes", I consent to use of the information provided in aggregate form only for this project.**

- ◯ Yes
- ◯ No

***2. In what year did you receive your first pharmacy degree?**

____

***3. What is your gender?**

- ◯ Female
- ◯ Male

***4. Which of the following best describes your current practice setting?**

- ◯ Independent Pharmacy (<4 stores under the same ownership)
- ◯ Small Chain Pharmacy (4-10 stores under the same ownership)
- ◯ Large Chain Pharmacy (>10 stores under the same ownership)
- ◯ Mass Merchandiser Pharmacy (e.g., Wal-Mart, Target, Kmart)
- ◯ Supermarket Pharmacy (e.g., Price Chopper, Hen House, Hy-Vee)
- ◯ None of the above

The next set of questions will ask you to recall your most recent encounter with a patient discharged from the inpatient hospital setting. Please answer as best you can remember.

***5. Thinking of your most recently discharged patient, how did you learn that he/she was discharged from the hospital? (select all that apply)**

- [ ] Patient or caregiver verbally told you
- [ ] Patient or caregiver gave you discharge papers
- [ ] Patient or caregiver asked a question about discharge information
- [ ] Information from written prescription
- [ ] Physician sent discharge information/paperwork
- [ ] I have never had a patient who was recently discharged from the hospital
- [ ] Other (please specify)

***6. Did you ask the patient/caregiver about other medication changes that may have been made for this patient?**

- ○ Yes
- ○ No

***7. If you did not ask about medication changes, what were the reasons? (select all that apply)**

- [ ] Lack of time
- [ ] Lack of staff
- [ ] Did not have an area to discuss this information privately
- [ ] This is beyond the scope of my position
- [ ] This is beyond the scope of my expertise
- [ ] Did not think to ask
- [ ] Other (please specify)

***8. Still thinking about your last discharged patient, what prescription problems were identified? (select all that apply)**

- [ ] Incomplete or unclear directions
- [ ] C-II prescription not valid
- [ ] Mismatch between quantity prescribed and days therapy
- [ ] Issues with prescriber identity or verification
- [ ] No prescription problems were identified
- [ ] Other (please specify)

***9. As best you can remember, how many prescription problems were identified for this patient? (please answer with a whole number "0, 1, 2, etc"**

***10. Still thinking about your last discharged patient, what medication-related problems were identified? (select all that apply)**

- [ ] Wrong drug
- [ ] Wrong dose/ route
- [ ] Drug interactions/adverse reactions
- [ ] Duplicate therapy
- [ ] Untreated indication for therapy
- [ ] Adherence (patient unable or unwilling to adhere to treatment)
- [ ] No medication-related problems were identified
- [ ] Other (please specify)

***11. How many medication-related problems were identified for this patient?**

***12. If the prescription or medication-related problems identified required contacting the prescriber, how many times did you attempt to do so?**

- [ ] I did not attempt to contact the prescriber
- [ ] Number of attempts to contact the prescriber:

***13. If you did not attempt to contact the prescriber, what were the reasons? (select all that apply)**

- [ ] Did not have accurate contact information
- [ ] Information is illegible
- [ ] No contact information provided by physician or prescriber
- [ ] Lack of time
- [ ] Lack of staff
- [ ] No need to contact as I was able to resolve on my own
- [ ] Other (please specify)

***14. If you were unable to reach the prescriber on the first attempt, what were the reasons? (select all that apply)**

- [ ] Did not have accurate contact information
- [ ] Hospital or clinic operator unable to directly connect to prescriber
- [ ] Information is illegible
- [ ] No contact information provided by physician or prescriber
- [ ] Could not reach outside of office hours
- [ ] Lack of time
- [ ] Lack of staff
- [ ] Other (please specify)

***15. In your best estimate, how many days did it take to receive a response from the prescriber? (if same day, enter "0"; if next day, enter "1", and so on)**

- ○ Did not receive response
- ○ Days it took to receive response from prescriber:

***16. For this recently discharged patient, did he/she receive all of the medications prescribed upon discharge?**

- ○ Yes
- ○ No

***17. If no, what were the reasons this patient did not get all discharge medications from the pharmacy? (select all that apply)**

- [ ] Medication was too expensive
- [ ] Prescription problem that could not be resolved at time of pick-up
- [ ] Insurance restrictions (e.g., prior authorization, step therapy, quantity limit)
- [ ] Patient did not expect a new medication
- [ ] Other (please specify)

***18. As best you can estimate, what percentage of the time is your pharmacy notified, by any means, when one of your patients is discharged from the hospital?**

- ○ 0-10%
- ○ 11-20%
- ○ 21-30%
- ○ 31-40%
- ○ 41-50%
- ○ 51-60%
- ○ 61-70%
- ○ 71-80%
- ○ 81-90%
- ○ 91-100%

***19. How important to optimal patient care is each of the following items?**

	Strongly disagree	Slightly disagree	Neither agree nor disagree	Slightly agree	Strongly agree
The patient's community pharmacist is notified, by any means, when one of their patients is discharged from the hospital.	◯	◯	◯	◯	◯
The patient's community pharmacist receives discharge documentation, including an updated medication list, when one of their patients is discharged from the hospital.	◯	◯	◯	◯	◯
Community pharmacists have access to information needed to identify medication and discharge errors in the community setting.	◯	◯	◯	◯	◯
Community pharmacists have access to information needed to answer patient questions about discharge plans and medications.	◯	◯	◯	◯	◯
Community pharmacists can directly contact the original prescriber to resolve medication or prescription problems identified for discharged patients.	◯	◯	◯	◯	◯
The community pharmacist notifies the patient's primary care physician when the patient does not receive discharge medications.	◯	◯	◯	◯	◯

20. We thank you for your time to complete this survey. Please provide any comments to address anything we may not have thought of below, if you desire.
